# A Chinese patient with developmental and epileptic encephalopathies (DEE) carrying a TRPM3 gene mutation: a paediatric case report

**DOI:** 10.1186/s12887-021-02719-8

**Published:** 2021-06-01

**Authors:** Qingyun Kang, Liming Yang, Hongmei Liao, Sai Yang, Xiaojun Kuang, Zeshu Ning, Caishi Liao, Bo Chen

**Affiliations:** Department of Neurology, Hunan Children’s Hospital, No.86 Ziyuan Road, Changsha, 410007 Hunan People’s Republic of China

**Keywords:** TRPM3, DEE, Seizure, Video-EEG, Case report

## Abstract

**Background:**

Developmental and epileptic encephalopathies (DEEs) are a heterogeneous group of chronic encephalopathies characterized by epilepsy with comorbid intellectual disability that are frequently associated with de novo nonsynonymous coding variants in ion channels, cell-surface receptors, and other neuronally expressed genes. Mutations in TRPM3 were identified as the cause of DEE. We report a novel patient with DEE carrying a de novo missense mutation in TRPM3, p.(S1202T); this missense mutation has never been reported.

**Case presentation:**

A 7-year and 2-month-old Chinese patient who had recurrent polymorphic seizures was clinically diagnosed with DEE. A de novo missense mutation in TRPM3, which has not yet been reported, was identified in this case. The patient had a clinical phenotype consistent with previous reports.

**Conclusions:**

These findings could expand the spectrum of TRPM3 mutations and might also support that de novo substitutions of TRPM3 are a cause of DEE.

## Background

Developmental and epileptic encephalopathies (DEEs) are a heterogeneous group of disorders characterized by the co-occurrence of epilepsy and intellectual disability (ID) [1]. DEE are thought to be largely caused by genetic factors [2], and most identified variants in individuals with DEE are in-frame, de novo, and recurrent across unrelated people [3].

Transient receptor potential (TRP) channels, which are predominantly localized to the plasma membrane, are a superfamily of gated cation channels sensitive to a variety of physical and chemical stimuli [4]. Seven subfamilies are recognized [5]. TRP channels are implicated in several disorders, including polycystic kidney disease, complete congenital stationary night blindness, familial hypomagnesemia with secondary hypocalcaemia, mucolipidosis type IV, amyotrophic lateral sclerosis-dementia-parkinsonism complex and others [5].

TRPM3 is expressed in peripheral sensory neurons, pancreatic B-cells, and vascular smooth muscle cells [6]. Moreover, TRPM3 is expressed in various regions of the brain, including the choroid plexus, cortex, cerebellum and hippocampal formation [7], but its functional role in these areas is essentially unexplored. A recent paper showed that two de novo missense mutations in TRPM3 (p. Val837Met and p. Pro937Gln) were associated with intellectual disability, hypotonia and epilepsy in eight probands with developmental and epileptic encephalopathy (DEE), indicating important roles of this channel in the human brain [8].

In this study, we report an individual carrying a TRPM3 gene mutation with a clinical phenotype of developmental and epileptic encephalopathies (DEE). This patient was heterozygous for a serine substitution, (p. Ser1202Thr), a missense mutation that has never been reported. We described the clinical features of the patient.

## Case presentation

**Fig. 1:**
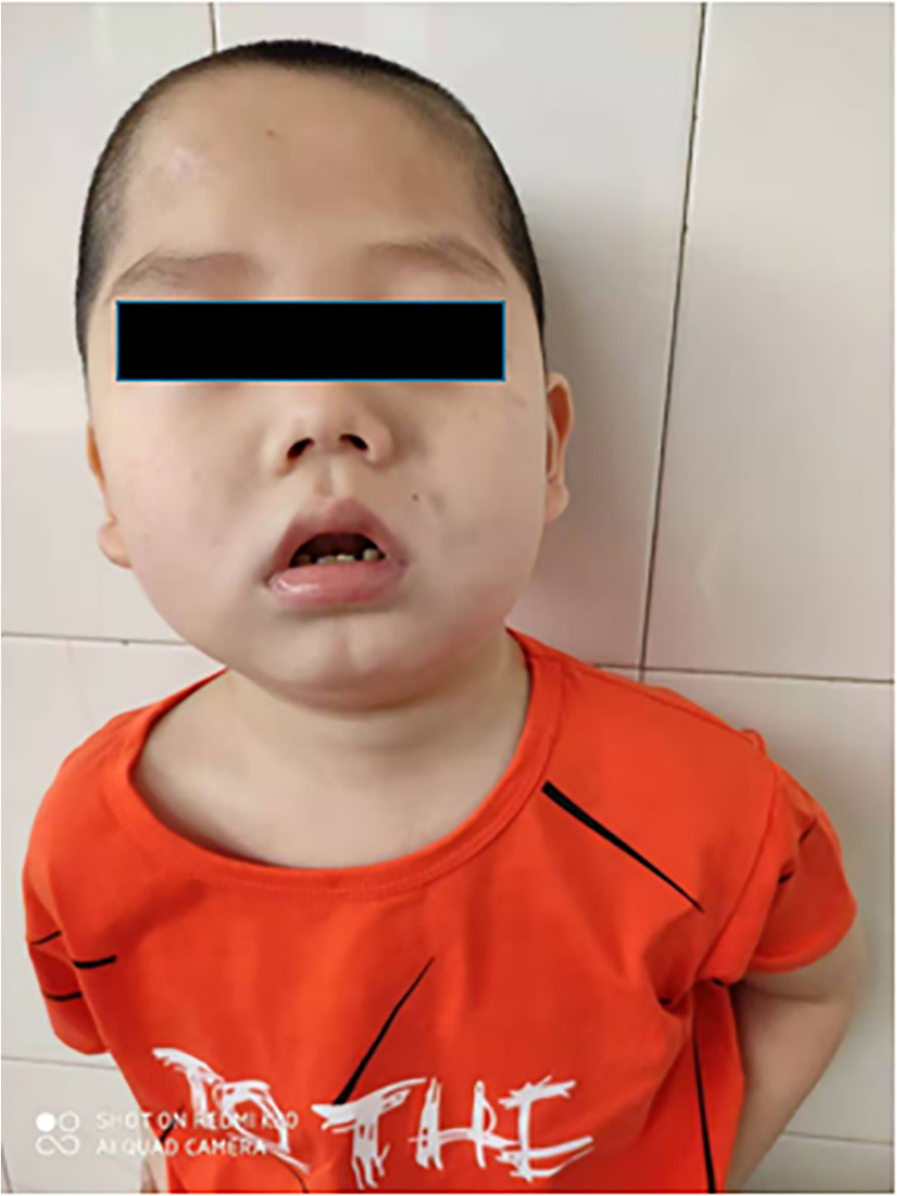
Facial appearance at 7 years and 2 months old. Several facial anomalies, such as broad forehead, short philtrum, micrognathia and prominent lobule of the ear, were observed. We obtained permission from the parents to post this photograph

A 7-year and 2-month-old Chinese boy was the second child of nonconsanguineous, healthy parents. The family history of the patient was unremarkable. The patient was born at 40 weeks of gestation with a birth weight of 3.6 kg, a body length of 50 cm and a head circumference of 31 cm. His motor development was slightly delayed. He began to gain head and neck stability at 4 months old and rolled over at 10 months old, and he could walk alone at the age of 18 months. His speech development was severely delayed. Several facial anomalies, including a broad forehead, short philtrum, micrognathia and prominent lobule of the ear, were observed (Fig. 1).

**Fig. 2:**
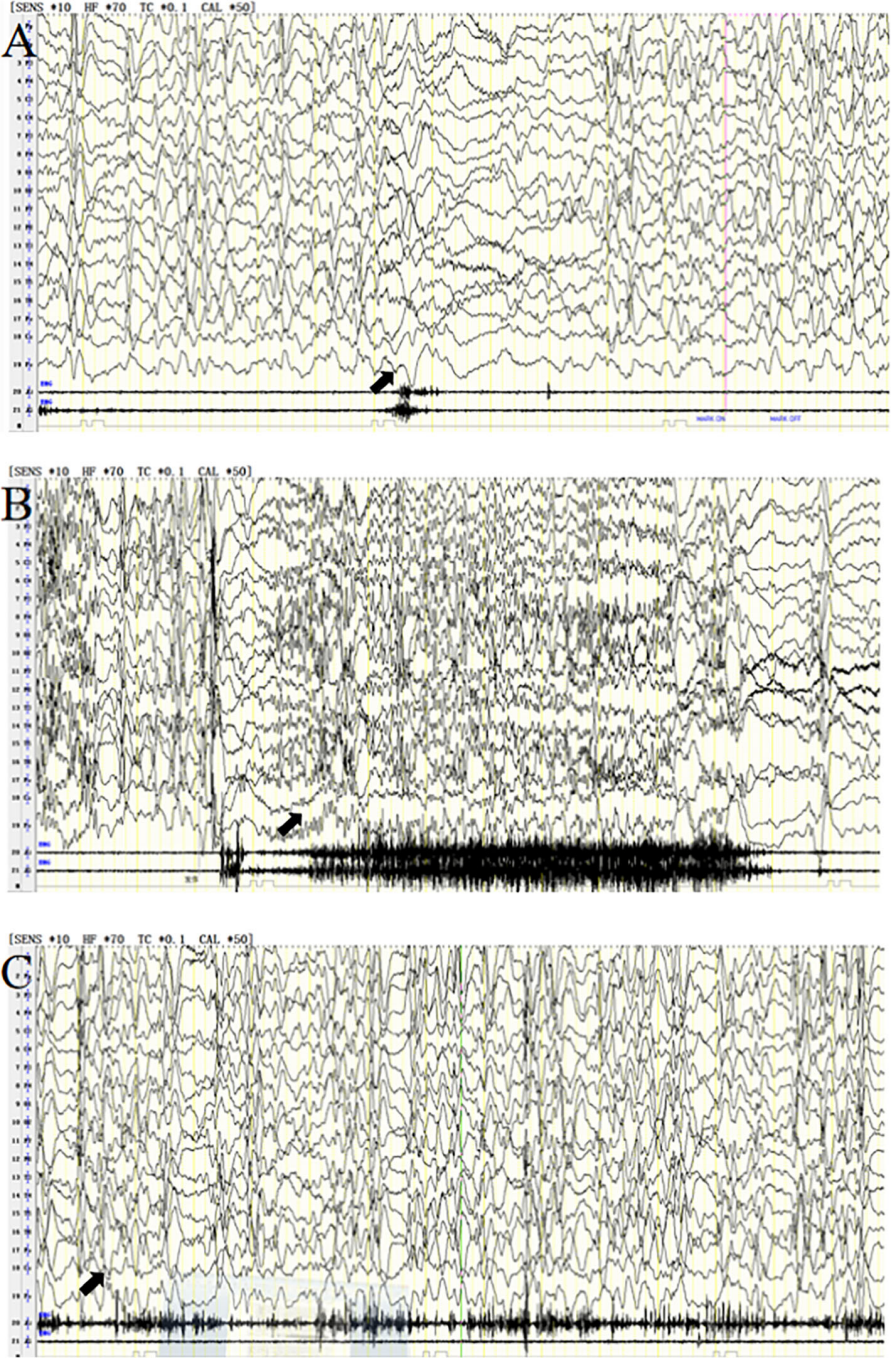
**A** EEG revealed typical epileptic spasm accompanied by generalized slow waves of high amplitude and fast waves of low amplitude. Hypsarrhythmic waves were observed during the interval between spasms. **B** EEG revealed tonic seizures accompanied by generalized spike wave rhythms of medium and high amplitude. **C** Atypical absence status epilepticus was monitored by video EEG

At the age of 9 months, the patient was occasionally observed to have paroxysmal shaking of his head and limbs with transient duration immediately after waking. Because there was no obvious abnormality in Electroencephalogram (EEG) and brain Magnetic Resonance Imaging (MRI), the observation was not considered serious, and no special treatment was given. At the age of 1 year and 10 months, the patient’s head nodding movements became increasingly obvious, and he was admitted to our department. A four-hour sleep-deprived EEG revealed hypsarrhythmia and typical epileptic spasms (Fig. 2A). Therefore, the patient was diagnosed with West syndrome. A slight expansion of the left lateral ventricle was observed in the brain MRI; however, no further abnormal findings were noted. Vitamin B6 was administered intravenously at a dose of 10 mg/kg.d for 7 days, but its effect was insufficient. Because adrenocorticotrophic hormone (ACTH) and Vigabatrin could not be acquired, vitamin B6 was substituted for a gradual titration of valproate (VPA) at an initial dose of 10 mg/kg.d, increasing to 30 mg/kg.d. The epileptic spasms were completely controlled over the following 8 months. However, 8 months later, epileptic spasms recurred in the patient, and tonic seizures were monitored by video EEG at the same time (Fig. 2B). Because ACTH treatment was refused by his parents, levetiracetam (LEV), at an initial dose of 10 mg/kg.d, increasing to 50 mg/kg.d, along with the previous VPA therapy were begun, but their effects were insufficient. One and a half month later, ACTH was administered intravenously; however, serious oedema prompted discontinuation of the treatment. Because the seizures were persistent, LEV was substituted for a gradual titration of both topiramate (TPM), at an initial dose of 1 mg/kg.d increasing to 8 mg/kg.d, and clonazepam (CZP), at an initial dose of 0.03 mg/kg.d increasing to 0.2 mg/kg.d. The seizures were gradually controlled 5 months later. By the time the patient was 3 year and 5 month old, epileptic spasms recurred, and, subsequently, a ketogenic diet was administered. Instead of the expected improvement, we observed an increase in the seizure frequency to 10 series per day during the waking period. Due to lack of efficacy,the parents refused the ketogenic diet 1 month later, and other antiepileptic drugs such as zonisamide or lamotrigine were also refused by the parents. VPA and TPM combined with CZP therapy were continued complying with medication regimen, but the seizures were out of control till now. Neurodevelopmental plateauing or regression was accompanied by frequent epileptiform activity. He had mild hypotonia and severe intellectual disability, at present, the patient can’t take care of himself and he can’t pronounce two meaning words until now. At the age of 7 years and 2 months, epileptic spasms, tonic seizures and atypical absence status epilepticus were monitored by video EEG (Fig. 2C), and V-EEG monitoring showed electrical status epilepticus during sleep (ESES). In this patient, changes in EEG monitoring eventually suggested Lennox-Gastaut syndrome (LGS).

### Molecular genetic analysis

**Fig. 3:**
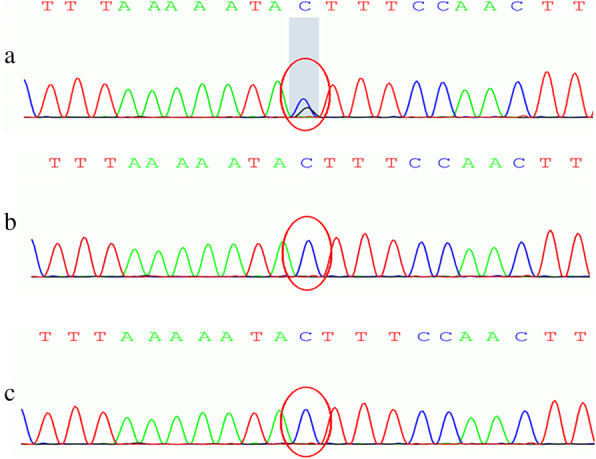
Sequencing of the TRPM3 gene: c.3605G > C (p.S1202T). The child has a heterozygous Mutation, and his parents are normal. (**a** patient; **b** the father; **c** the mother)

Chromosomal microarray analysis (CMA)、hole-exome sequencing (WES) and Sanger sequencing were performed by Running Gene Inc. (Beijing, China) using their standard process, which is available in a previous report [9]. In our case, the CMA was normal, the variant c.3605G > C (GRCh37/ hg19, NM_020952) of TRPM3 (Fig. 3) was identified in the patient, the mutation was absent in the parents, suggesting that this mutation was de novo. This variation would cause a substitution, p.S1202T. Since this variation is a de novo variation carried by neither of the parents (PS2) and is also a variation that is absent from controls (PM2), it was classified as “likely pathogenic” according to the ACMG guidelines.

## Discussion and conclusion

Dyment et al. [8] recently reported on eight novel patients with epilepsy and intellectual disability associated with heterozygous de novo missense variants in TRPM3. This was the first report to show that de novo missense mutations of TRPM3 were associated with developmental and epileptic encephalopathy (DEE). Among the eight probands, seven patients across multiple unrelated kindreds showed a consistent clinical phenotype that was identically heterozygous for a recurrent substitution, p. (Val837Met). The eighth proband showed a similar clinical phenotype and was heterozygous for a proline substitution, p. (Pro937Gln). Therefore, the two de novo heterozygous mutations in the gene encoding TRPM3 were identified as the cause of DEE in the eight probands. Our patient also had a missense variant in TRPM3, and the mutation of TRPM3 carried in our patient was p.(S1202T), which has not yet been reported.

The 8 probands [8] all had moderate-to-severe global developmental delay, hypotonia and seizures (1 with epileptic spasm). The patient in our study had a clinical phenotype consistent with the previous descriptions. He showed severe global developmental regression associated with frequent epileptiform activity; he could not go to the toilet independently and had no speech until now. The facial features of the patient (Fig. 1) are similar to those of the previous 8 probands [8]. The patient has a broad forehead, short philtrum, micrognathia and prominent lobule of the ear; a micropenis was also observed. Therefore, we propose that de novo substitutions of TRPM3 are the cause of ID and epilepsy in our patient.

**Fig. 4:**
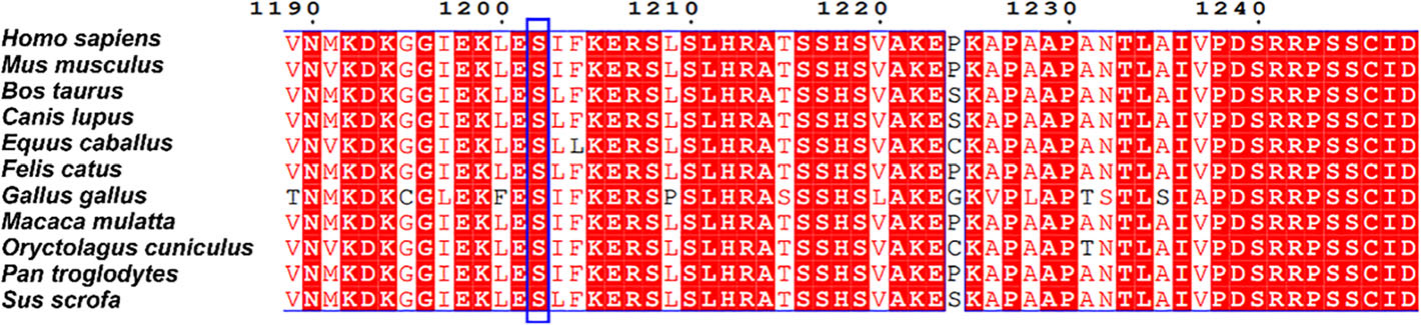
Sequence alignment of TRPM3 proteins from different species. Residue S1202 is highly conserved (indicated by a blue box)

However, the mechanism of mutations in the TRPM3 gene causing DDE has not been established. Recently, two studies [10, 11] reported that mutations p.V990M (S4-S5 loop) and p.P1090Q (extracellular segment of S6) resulted in a profound gain of channel function of TRPM3, which increased inward cation currents and Ca^2+^ influx, causing epileptic activity and neurodevelopmental symptoms in patients. However, in our study, the detected mutation p.S1202T located in the cytoplasmic segment, which was different from these reported mutations. S1202 was highly conserved in different spices (Fig. 4), and was predicted to be damaging (MutationTaster score 0.891), possibly damaging (PolyPhen-2 HDIV score 0.447). Substitution of S1202 might result in abnormally high calcium levels, damage nerve cells and finally cause disease. Further experiments were needed to study whether the mutation could also result in gain of function or other effects on TRPM3. The epilepsy drug primidone switched off the mutant channels, pointing to potential treatment of this disease using primidone [10, 12]. Although many therapeutic methods have been applied to control epileptiform activity, there were no effects, and our patient still presents several kinds of seizures simultaneously. Since last week, we have been trying to treat our patient with primidone, but the effect is still under observation.

TRPM3-related DEE is rare, thus far, only eight patients have been reported. By reporting a case of DEE caused by a previously unreported TRPM3 mutation, we have expanded on what is known about the variety of TRPM3 mutations, thus laying the foundation for future research.

## Data Availability

If you need more data or materials about this case, please contact the corresponding author Dr. Bo Chen.

## Abbreviations

DEE: Developmental and epileptic encephalopathies
TRPM3: Transient Receptor Potential Melastatin 3
ID: Intellectual disability
TRP: Transient receptor potential
EEG: Electroencephalogram
MRI: Magnetic resonance imaging
ACTH: Adrenocorticotropic hormone
VPA: Valproate
LEV: Levetiracetam
CZP: Clonazepam
TPM: Topiramate
ESES: Electrical status epilepticus during sleep
LGS: Lennox-Gastaut syndrome
WES: Whole-exome sequencing.

## References

[1] Scheffer IE, Berkovic S, Capovilla G (2017). ILAE classification of the epilepsies: position paper of the ILAE Commission for Classification and Terminology. Epilepsia..

[2] Happ HC, Carvill GL (2020). A 2020 view on the genetics of developmental and epileptic Encephalopathies. Epilepsy Curr.

[3] Hamdan FF, Myers CT, Cossette P, Lemay P, Spiegelman D, Laporte AD (2017). High rate of recurrent de novo mutations in developmental and epileptic encephalopathies. Am J Hum Genet.

[4] Farooqi AA, Javeed MK, Javed Z (2011). TRPM channels: same ballpark, different players, and different rules in immunogenetics. Immunogenetics..

[5] Nilius B (1772). TRP channels in disease. Biochim Biophys Acta.

[6] Vriens J, Owsianik G, Hofmann T (2011). TRPM3 is a nociceptor channel involved in the detection of noxious heat. Neuron.

[7] Grimm C, Kraft R, Sauerbruch S (2003). Molecular and functional characterization of the melastatin-related cation channel TRPM3. J Biol Chem.

[8] Dyment DA, Terhal PA, Rustad CF (2019). De novo substitutions of TRPM3 cause intellectual disability and epilepsy. Eur J Hum Genet.

[9] Wang X, Shen X, Fang F, Ding CH, Zhang H, Cao ZH (2018). Phenotype- driven virtual panel is an effective method to analyze WES data of neurological disease. Front Pharmacol.

[10] Zhao S, Yudin Y, Rohacs T (2020). Disease-associated mutations in the human TRPM3 render the channel overactive via two distinct mechanisms. Elife..

[11] Hoeymissen EV, Held K, Nogueira Freitas AC (2020). Gain of channel function and modified gating properties in TRPM3 mutants causing intellectual disability and epilepsy. Elife..

[12] Krügel U, Straub I, Beckmann H (2017). Primidone inhibits TRPM3 and attenuates thermal nociception in vivo. Pain..

